# Booze, Boozer, Boozy

**Published:** 1911-11

**Authors:** 


BOOZE, BOOZER, BOOZY.—Few of my readers, I apprehend, are aware that these words are classical, but think as I did before I came across "booze" recently in Horace and looked them up. Horace says that Homer was a boozer. The words are, therefore, of very ancient origin and were known to the Greeks and Romans,—the etymology is obscure and uncertain. Certain it is they belong now to the vernacular and are generally regarded as slang. Hence their use is avoided by the polite, in the presence of the cultured, the fastidious preferring to say "intoxicated," using the word—as Webster does, as synonymous with "boozy." Landon also uses it, and Haweis and Chas. Kingsley and even Walter Scott. When a man is "poisoned" (with liquor) we say he is "intoxicated," which the word means, and we laugh at his antics and foolish behavior and the subject is one of no end of jokes. We arrest him, perhaps lock him up and put him to breaking stones on the street, or fine him. Were he "intoxicated" with hyoscyine, or *coculus indica,* or other poison we would send him to the hospital for treatment. I see nothing "funny" in a drunken man. To me it is pitiful; a pathetic sight; certainly nothing to laugh at. To see a young man drunk is painful in the extreme; to see an old man drunk is truly distressing. They are poisoned; they are sick men and should be so treated. It is not so long ago that the insane were treated as criminals, beaten, locked up and perhaps put to work, as we now put the poor drunkard to work. But it would "injure business"; it would interfere with personal rights, if we did not permit him to poison himself. Pity, pity.

